# Exploring Family Quality of Life Among Saudi Families of Children with Autism Spectrum Disorder

**DOI:** 10.3390/healthcare14193233

**Published:** 2026-10-01

**Authors:** Mohammed Al Jaffal

**Affiliations:** Department of Special Education, College of Education, King Saud University, Building 15, Ash-Shaikh Hasan Ibn Abdullah Al Ash-Shaikh, Riyadh 12372, Saudi Arabia; moaljaffal@ksu.edu.sa

**Keywords:** autism spectrum disorder, family quality of life, parents, qualitative research, family adaptation

## Abstract

**Highlights:**

**What are the main findings?**

- Family quality of life was influenced by caregiving demands, access to effective services, family adaptation, children’s progress, and social support.
- Families valued services that responded to their children’s needs and reported that children’s progress improved parents’ confidence and hope.

**What are the implications of the main findings?**

- Families need continuous, family-centered services that respond to their children’s changing needs.
- Better community understanding and inclusive education can support family well-being and participation in everyday life.

**Abstract:**

Background/Objectives: Family quality of life (FQOL) among families of children with autism spectrum disorder (ASD) is influenced by family life, services, and social experiences. Although research in Saudi Arabia has examined caregiver well-being and services, less is known about how families experience FQOL in everyday life. This study explored the experiences and perceptions of Saudi parents and caregivers of children with ASD. Methods: A qualitative descriptive design was used. Semi-structured interviews were conducted with 12 Saudi parents and caregivers of children with ASD. Data were analyzed using thematic analysis, with the FQOL framework used as a sensitizing conceptual lens. Results: Five themes were identified: (1) Diagnostic Experiences and Family Reorientation; (2) Caregiving and Family Reorganization; (3) Responsive Services and Family Quality of Life; (4) Family Adaptation Through Knowledge and Resilience; and (5) Community Understanding and Social Inclusion. Findings showed that FQOL was influenced by caregiving demands, responsive services, family adaptation, child progress, and opportunities for social participation. Conclusions: FQOL is shaped by families’ changing experiences and needs over time. Supporting Saudi families requires responsive services, family-centered support, continuity across service systems, and more inclusive communities.

## 1. Introduction

Autism spectrum disorder (ASD) is a neurodevelopmental condition characterized by persistent differences in social communication and interaction, alongside restricted and repetitive patterns of behavior, interests, or activities [1]. Although these characteristics are expressed at the individual level, their implications often extend into family life. Families may navigate changing developmental and behavioral needs, decisions regarding intervention and education, coordination of services, and concerns about long-term support and independence. Understanding outcomes in autism therefore requires attention not only to the individual with ASD but also to the well-being and functioning of the family as a whole.

Family Quality of Life (FQOL) provides a useful framework for understanding these experiences. Rather than focusing exclusively on individual outcomes such as parental stress, caregiver burden, or psychological well-being, FQOL considers the family as the primary unit of analysis and recognizes multiple interconnected dimensions of family life, including family interaction, parenting, emotional well-being, physical and material well-being, and disability-related support [2,3]. FQOL has also been conceptualized as a dynamic outcome emerging from interactions among individual and family characteristics, available resources and supports, and broader environmental conditions [4]. This perspective is particularly relevant to families of children with ASD because their priorities and support needs may change as children develop and caregiving responsibilities evolve.

International research supports this multidimensional understanding of FQOL. Families of children with ASD may experience changes in routines, relationships, employment, leisure, and other aspects of everyday family life, although these experiences vary according to available personal, family, and environmental resources [5,6]. Social support, coping, self-efficacy, and resilience appear to be particularly relevant to family adaptation. A meta-analytic structural equation modeling study involving 29 studies and 4864 caregivers found that coping was associated with FQOL both directly and indirectly through social support [7]. Longitudinal evidence has similarly shown that parenting self-efficacy and social support are associated with higher FQOL among parents of children newly diagnosed with ASD and that FQOL may change over time [8]. Resilience has also been identified as an important resource associated with family quality of life among parents of children with ASD [9]. These findings suggest that adaptation is an evolving process shaped by interactions between family challenges and available resources.

Formal services constitute another important part of this process. Families may interact with diagnostic, educational, therapeutic, healthcare, and community-based systems over extended periods. However, service availability does not necessarily ensure that families experience support as effective. Accessibility, continuity, intensity, quality, responsiveness to individual needs, and families’ ability to navigate service systems may all shape their experiences. When services are difficult to access or poorly matched to children’s needs, families may assume additional coordination, financial, and advocacy responsibilities. Conversely, responsive support may contribute to improvements in child functioning and reduce demands on everyday family life [5,9].

Social and community environments are similarly important. Opportunities for family participation may depend partly on whether relatives, professionals, schools, and community members understand and respond appropriately to autism. Social support can provide practical and emotional resources, whereas stigma and misunderstanding may create additional demands. FQOL therefore reflects not only processes within the household but also families’ interactions with the wider environments in which everyday life occurs [4,10].

These factors have received increasing empirical attention in Saudi Arabia. Earlier research identified substantial service-related challenges and variation in service utilization according to family income, parental education, autism knowledge, and geographic location [11]. Social factors have also emerged as important. Khusaifan and El Keshky [12] found that social support played a protective role in the relationship between parental stress and life satisfaction among Saudi parents of children with ASD, while Alshaigi et al. [13] documented experiences of autism-related stigma among Saudi parents. More recently, Salami and Alhalal [14] identified affiliate stigma, perceived social support, and family functioning as important correlates of parental quality of life. These studies indicate that family well-being in Saudi Arabia is shaped by interacting individual, family, service, and social conditions.

Recent Saudi research has increasingly examined quality of life, although at different levels of analysis. Alhuzimi [15] examined parents’ perceptions of factors influencing the quality of life of their children with ASD and identified children’s difficulties, family support, services and interventions, and family and child characteristics as relevant factors. In contrast, Alnahdi et al. [16] examined FQOL directly using the Arabic version of the Beach Center Family Quality of Life Scale in a sample of 1065 family members, identifying challenges across domains including family interaction, parenting, emotional well-being, and physical/material well-being. A more recent Saudi study of 302 caregivers using the WHOQOL-BREF further documented physical, psychological, social, and environmental dimensions of caregiver quality of life and the contribution of demographic circumstances to these experiences [17]. These studies highlight an important conceptual distinction: child quality of life, individual caregiver quality of life, and FQOL as a collective family construct are related but distinct outcomes.

Saudi research has also drawn attention to the quality and responsiveness of autism services. Almasoud and Ain [18], comparing parental perspectives across 2011 and 2021, reported improvements in autism services alongside challenges that persisted across the decade. Almasoud and Alqahtani [19] similarly found that parents connected the quality of diagnostic, early-intervention, educational, and healthcare services with quality-of-life outcomes for individuals with ASD and their families. Recent evidence has identified variation in parental satisfaction across guidance, evaluation, intervention and support, and services intended to enhance quality of life [20]. These findings suggest that families may distinguish between the availability of services and their actual accessibility, quality, and responsiveness to individual and family needs.

Qualitative evidence provides an important complement to these findings. Hemdi and Daley [21], through interviews with Saudi mothers of children with ASD, identified needs related to autism knowledge, parental well-being, continuing caregiving demands, parent training, children’s education, and public awareness. Such work demonstrates the value of allowing families to describe experiences that may not be fully captured through predetermined quantitative measures and indicates that family experiences extend across multiple areas of everyday life.

Despite this growing evidence base, an important gap remains. Saudi research has largely examined particular components of family experience separately, including parental stress and life satisfaction, child quality of life, caregiver quality of life [12,14,15,17], standardized FQOL domains [16], service access and utilization [11], and perceptions and satisfaction of service quality [18,19,20]. Qualitative research has provided valuable insight into particular aspects of family experience, especially maternal needs [21], but less is known about how Saudi parents understand FQOL as a multidimensional and evolving family experience and how different aspects of family life become interconnected.

This gap is important because families may not experience diagnosis, caregiving, service use, adaptation, and social participation as independent domains. These experiences may interact as families adjust responsibilities and expectations, develop knowledge and caregiving competence, navigate formal and informal supports, and respond to changes in their children’s development and social environments. A qualitative examination can therefore complement existing quantitative FQOL evidence by exploring how parents themselves interpret relationships among these experiences.

The specific contribution of this study is therefore to provide an integrated qualitative account of how Saudi families themselves understand the connections among diagnosis, caregiving, service experiences, adaptation, and social participation as components of an evolving family quality-of-life experience. Accordingly, the present study aimed to explore Saudi parents’ perceptions and experiences of FQOL while raising children with ASD. Guided by the FQOL framework while remaining open to patterns emerging from participants’ accounts, the study examined how parents describe their family quality of life, the factors they perceive as shaping it, and the sources of support and adaptation they draw upon within the Saudi sociocultural context.

**Research Questions:** How do families of children with ASD in Saudi Arabia describe their quality of life? What factors shape the quality of life of families of children with ASD? What sources of support and coping do families utilize to enhance their quality of life?

## 2. Materials and Methods

### 2.1. Research Design

This study employed a qualitative descriptive design to explore how Saudi parents of children with ASD experience and understand FQOL. This approach was selected because it enables rich yet relatively straightforward accounts of participants’ experiences while remaining close to their perspectives and meanings.

The study was informed by the FQOL framework, which conceptualizes family well-being as multidimensional and shaped by family relationships, emotional and physical well-being, material resources, support, services, and participation. The framework informed the interview protocol and served as a sensitizing conceptual lens during analysis rather than as a predetermined structure for the findings.

### 2.2. Participants

Participants were 12 parents or primary caregivers of children diagnosed with ASD residing in Saudi Arabia (Table 1). Purposeful sampling was used to recruit participants who could provide rich and meaningful information regarding family experiences associated with raising a child with ASD. Inclusion criteria required participants to: (a) be a parent or primary caregiver of a child formally diagnosed with ASD, (b) reside in Saudi Arabia, and (c) be willing to participate in an individual interview. Although most participants were parents, one participant (P6) was the sister of an adult with ASD (aged 24) and served as her primary caregiver. She therefore met the study’s inclusion criterion of being a parent or primary caregiver. Given her different caregiving role, her account was analyzed using the same thematic process as that of the other participants. Her experiences contributed to the themes where relevant but did not independently shape or alter their development.

To maximize variation in perspectives, efforts were made to recruit participants representing different geographic regions, educational backgrounds, socioeconomic levels, and child characteristics. Participants represented the Central, Northern, Southern, Eastern, and Western regions of Saudi Arabia, with some residing in smaller cities and villages. They also represented a range of educational and socioeconomic backgrounds. Specific geographic locations are not reported to protect participant confidentiality. Recruitment and data collection continued until the dataset was considered sufficiently information-rich to address the research questions and no substantively new patterns relevant to the developing themes were identified in the later interviews. This judgment was made by the author based on the data collected and the patterns identified during analysis.

### 2.3. Data Collection

Data were collected through semi-structured individual interviews conducted by the researcher. The interviews were conducted between 24 June and 6 July 2026, following ethical approval on 15 June 2026. The interview protocol was informed by the FQOL framework and relevant literature on families of children with ASD. Questions explored family relationships, psychological and physical well-being, everyday family life, service experiences, financial challenges, social interactions and community attitudes, coping and adaptation, and perceptions of overall family quality of life.

Interviews were conducted using Zoom based on participants’ preferences and convenience, allowing them to participate from a private and comfortable setting. Interviews lasted approximately 45 min. With participants’ permission, interviews were audio-recorded and transcribed for analysis. Interviews were conducted and transcribed in Arabic. Selected quotations were translated into English by the author and subsequently reviewed by a translator holding a master’s degree in translation. The translations were checked against the original Arabic transcripts to ensure that participants’ intended meanings were preserved. Before data collection, the interview protocol was reviewed by experts in special education and qualitative research for clarity, relevance, and alignment with the study objectives, and minor revisions were made based on their feedback.

### 2.4. Reflexivity

Given the researcher’s professional background in special education and autism, reflexivity was maintained throughout data collection and analysis. Reflective memos were used to document prior assumptions, emerging interpretations, and potential influences on engagement with the data. These reflections were revisited during analysis to distinguish participants’ accounts from the researcher’s professional assumptions and to support interpretations grounded in participants’ experiences. For example, the researcher initially expected service-related concerns to focus primarily on service availability. Reflexive memoing helped make this assumption explicit and allowed the coding to remain responsive to participants’ emphasis on service responsiveness and changing needs.

### 2.5. Data Analysis

Interview recordings were transcribed verbatim and analyzed using Braun and Clarke’s [22] six-phase thematic analysis. All transcripts were coded by the author using NVivo, consistent with the study’s interpretive approach, rather than through inter-coder agreement. Codes were compared across transcripts and gradually grouped into potential themes. A hybrid inductive–deductive approach was employed. Coding remained primarily data-driven, while the FQOL framework served as a sensitizing conceptual lens during coding refinement and interpretation. This approach allowed the analysis to remain conceptually informed without restricting findings to predetermined FQOL domains.

Analysis proceeded through six phases: (1) familiarization with the data through repeated reading of transcripts; (2) generation of initial codes; (3) development of potential themes; (4) review and refinement of themes; (5) definition and naming of themes; and (6) production of the final report. Throughout the process, transcripts were revisited and patterns were compared across participants to identify both shared and divergent experiences. Representative quotations were selected to illustrate the themes and preserve participants’ voices.

### 2.6. Trustworthiness

Trustworthiness was addressed through strategies related to credibility, dependability, confirmability, and transferability. Credibility was supported through sustained engagement with the interview data, follow-up questioning during interviews, and participant verification. Four participants who were available and willing to provide follow-up feedback were invited to review written summaries highlighting the main points from their interviews. They were asked to confirm whether the summaries accurately reflected their experiences and to identify any points requiring clarification or correction. No substantive corrections were requested. Dependability was strengthened through an audit trail documenting key methodological and analytic decisions, including coding and theme development. Confirmability was supported through reflexive memoing and systematic documentation of analytic decisions. Transferability was facilitated through detailed descriptions of the study context, participants, procedures, and family experiences, enabling readers to assess the relevance of the findings to other contexts.

### 2.7. Ethical Considerations

Ethical approval was obtained from King Saud University board before data collection. Participants received information about the purpose of the study, the voluntary nature of participation, confidentiality, and their right to withdraw at any time without penalty. Written informed consent was obtained before participation. Identifying information was removed from transcripts, and participant codes were used in the reporting of findings to protect confidentiality.

## 3. Results

Analysis of the interviews identified five main themes describing families’ experiences of raising a child with autism and the factors shaping their family quality of life. These themes and their related subthemes are presented in Table 2 and described in the following sections.

### 3.1. Theme 1. Diagnostic Experiences and Family Reorientation

Participants described diagnosis as a process that developed over time rather than a single event. Families first noticed differences in their children’s development and then sought explanations and professional assessment. Receiving the diagnosis changed how parents understood their children and began to think about their needs and future.

#### 3.1.1. Early Recognition of Developmental Differences

Families often noticed developmental differences before autism was formally identified. Parents described differences in eye contact, communication, social interaction, and behavior, although they did not always understand what these signs meant at the time. One mother of twins (P5), for example, recalled noticing difficulties with eye contact and awareness of danger. Another mother described being particularly concerned about her child’s strong attachment to routines and his intense reactions when those routines changed: “I was very concerned about his routines and how extremely upset he became whenever they changed.” (P1). These early observations led some parents to seek further explanations and professional assessment.

#### 3.1.2. Diagnostic Uncertainty and Delayed Identification

Obtaining a diagnosis was not always straightforward. Some families described visiting different hospitals, specialists, and centers while seeking explanations for their children’s development. In some cases, parents received different professional opinions, including suggestions of language or developmental delay. This uncertainty made it difficult for families to know what support their children needed.

Access to specialized services also affected the diagnostic experience. One participant (P6) described her sister’s childhood in a region where autism services were limited and felt that earlier access to assessment and intervention could have made a difference. Other parents similarly described seeking additional opinions before reaching a clearer understanding of their children’s needs.

#### 3.1.3. Emotional Responses and Family Reorientation

Receiving the diagnosis was emotionally difficult for many families. Participants described feelings of shock, sadness, fear, and uncertainty about their children’s future. One mother described how her feelings changed over time: “It was difficult… but after a while I said, ‘That’s it… I have to accept it, because I’m the one who has to help them.’” (P5). For some parents, acceptance developed gradually and helped them focus more on what they could do for their children. Another mother explained: “At first, I kept asking myself why… but later I realised that what mattered was knowing what I could do for him.” (P3).

### 3.2. Theme 2. Caregiving and Family Reorganization

Following diagnosis, caregiving became an important part of everyday family life. Participants described adjusting their routines, responsibilities, work, and personal time around their children’s needs. Over time, families developed different ways of sharing responsibilities and maintaining stability while responding to their children’s changing needs.

#### 3.2.1. Continuous Caregiving Responsibilities

Participants described caregiving as an ongoing part of everyday family life. Daily routines often involved therapy, school, behavioral support, and supervision, requiring parents to organize much of their time around their children’s needs. For some families, these responsibilities continued as their children grew older. One participant (P6), who cared for her adult sister with autism, described how the family shared responsibility for her care: “We divided the responsibility among ourselves… each of us takes care of her for a certain period, because one person cannot manage everything alone.” (P6).

#### 3.2.2. Family Roles and Shared Responsibility

Caregiving also changed how responsibilities were shared within families. Participants described adjusting routines and sharing responsibilities according to their child’s needs. Over time, some families became more cooperative in managing caregiving. One mother explained: “We learned how to cooperate and share responsibilities better. The family became more aware of my child’s needs and how to deal with him.” (P7). Parents also emphasized the importance of being consistent in how they responded to their child. One mother explained: “We have to be consistent… we cannot have one person respond to him one way and the other respond differently.” (P3).

#### 3.2.3. Balancing Caregiving with Personal and Family Life

Caregiving responsibilities also affected participants’ personal time and work. Several participants described organizing their daily lives around their child’s needs, often leaving limited time for themselves. For some, balancing caregiving with work and other responsibilities was difficult. One mother explained: “My child’s needs were always my priority, so the time I had for myself was very limited.” (P8).

Participants also described adjusting their routines and personal plans to ensure that appropriate care was available. Despite these demands, families tried to maintain stability and positive relationships at home. Over time, some families learned to work together, share responsibilities, and better understand the child’s needs. One mother explained: “The experience strengthened our patience and cooperation as a family. We became more aware and more supportive of one another.” (P12).

### 3.3. Theme 3. Responsive Services and Family Quality of Life

Access to appropriate services emerged as an important influence on family quality of life. Participants emphasized that the value of services depended not only on their availability, but also on how well they responded to their children’s individual needs. Families particularly valued professional expertise, continuity of intervention, communication with parents, and observable improvements in their children.

#### 3.3.1. Service Responsiveness and Perceived Effectiveness

Participants emphasized that access to services did not always lead to positive outcomes. Parents often evaluated services based on the changes they observed in their children and, when necessary, moved between centers to find more appropriate support. One mother described a clear difference between two centers: “I enrolled him in a centre… honestly, within only two or three weeks, he regressed significantly. They would give him the phone and turn on the television… After we moved him to another centre, he changed completely. His sleep improved, his communication improved, and he started following instructions.” (P1). This experience shows that parents valued services based on how well they responded to their child’s needs and whether they produced noticeable improvements. The amount of intervention was also important. One participant felt that the publicly supported services available to her child were not enough: “My son receives only one session a week… and I know he needs more.” (P2). For some families, limited intervention led them to seek additional private services when they could afford them.

#### 3.3.2. Changing Needs and Continuity of Support

Participants also described difficulties when children’s developmental progress changed their eligibility for particular services. One father explained that his son had progressed beyond the admission criteria of some specialized centers, yet mainstream educational settings were not sufficiently prepared to accommodate his remaining needs: “He no longer needs a rehabilitation centre… but at the same time, the regular school is not prepared for him. We are caught between the two.” (P9). This created a gap in support for some families. As children developed, their need for support did not necessarily disappear; instead, the type of support they needed changed. Families therefore emphasized the importance of services that could respond to children’s changing needs over time.

#### 3.3.3. Barriers to Service Access and Financial Pressure

Families described several barriers to accessing appropriate services, including where they lived, limited availability of specialized programmes, and waiting periods. For some families, these difficulties were greater when their children were younger and fewer services were available. One participant explained: “She entered centers when she was older… When she was young, the situation was different. We did not have places to take her to.” (P6).

Financial pressure was another challenge for families. Participants valued the government-funded services and support available to their children and described them as important sources of assistance. However, they also noted that caring for a child with autism could involve additional costs, particularly when families sought private services beyond those provided or funded by public agencies. These included additional therapy, assessments, and other specialized services. One participant explained: “Everything costs… even the monthly assessments have a price, and if we need a hospital or private treatment, it is expensive but we must provide it for my child.” (P1). The participant was referring specifically to assessments obtained through private-sector services.

#### 3.3.4. Child Progress and Family Quality of Life

Parents described their children’s progress as an important way in which services influenced family quality of life. Improvements in communication, behavior, independence, and everyday functioning were described as benefiting not only the child but also the family. One mother reflected on the progress of her twins after several years of intervention: “After three continuous years of sessions, there was a big difference… especially ABA and occupational therapy; they had a great impact.” (P5).

For some parents, seeing their child make progress also gave them hope and reassurance that their efforts were making a difference. One participant explained: “Whenever I see her developing, I feel that our efforts have not been wasted.” (P4).

### 3.4. Theme 4. Family Adaptation Through Knowledge and Resilience

Participants described family adaptation as a gradual process that developed over time. As families learned more about autism and gained experience in supporting their children, they became more confident in understanding and responding to their needs. Knowledge, acceptance, and experience helped families manage challenges and maintain hope for their children’s future.

#### 3.4.1. Acceptance as a Foundation for Adaptation

Participants described acceptance as an important part of adapting to their child’s diagnosis. Although feelings of shock, sadness, and uncertainty did not immediately disappear, parents gradually shifted their attention from asking why autism had occurred to understanding their child’s needs and how they could support them. One mother of twins explained: “It was difficult… but after a while I said, ‘That’s it… I have to accept it, because I’m the one who has to help them.’” (P5). Another participant described a similar change over time: “At first, I kept asking myself why… but later I realised that what mattered was knowing what I could do for him.” (P3). For these parents, acceptance helped them focus more on their children’s needs and the support they could provide.

#### 3.4.2. Knowledge as a Source of Empowerment

Learning about autism was an important part of participants’ adaptation. Parents described gaining knowledge through professionals, personal reading, training, and their own experiences with their children. This helped them better understand their children’s behavior and respond with greater confidence. One participant explained: “When I started understanding autism, many things changed for me… I began to understand why he behaved in certain ways.” (P3).

For some parents, this search for knowledge went further and led them to pursue training in Applied Behavior Analysis (ABA) to better support their children. One mother explained: “I entered the field for his sake… the more I learned, the more I felt that I could understand and help him, and later I started helping other children as well.” (P3). A similar experience was described by P1, who also pursued ABA training to better understand and support her child.

### 3.5. Theme 5. Community Understanding and Social Inclusion

Participants described FQOL as being influenced by their experiences with relatives, schools, healthcare providers, and the wider community. Families valued greater understanding and acceptance of autism and wanted their children to participate in everyday life without judgment or unnecessary barriers.

#### 3.5.1. Public Understanding of Autism

Participants described limited public understanding of autism as a challenge in everyday family life. Children’s behaviors were sometimes interpreted by relatives or others as poor discipline or inadequate parenting rather than understood in the context of autism. Parents therefore often found themselves explaining their children’s behavior and correcting misconceptions. One mother explained: “They used to tell me, ‘Why don’t you let him hear what we’re saying? He needs to learn.’ They didn’t understand that he was overwhelmed.” (P8). Another participant described a similar experience: “They would say, ‘Just be firm with him… you’ve spoiled him too much.’ They didn’t understand that he was different from other children.” (P10). Such experiences showed how limited understanding of autism could add another challenge to families’ everyday interactions.

#### 3.5.2. Community Participation and Support

Families described participation in social and community life as being influenced by how well others understood and accommodated their children. Visiting relatives, travelling, and attending social gatherings sometimes required additional preparation. One participant explained: “We used to travel with him… but we had to make sure that the people around us knew how to deal with him.” (P7). Another participant described how greater understanding from relatives made social participation easier: “At first, I avoided social gatherings… but over time, when they started to understand him, things became much easier for me.” (P11). Families also valued practical support from relatives who understood their child’s needs. For one mother, her sister regularly cared for her child while both parents were at work, as she did not feel comfortable leaving him alone with the family’s helper. She particularly valued this support because her sister understood her child and knew how to care for him.

#### 3.5.3. Meaningful Inclusion

Participants described inclusion as more than simply being present in the community or school. They wanted their children to be understood, accepted, and supported rather than treated with sympathy. One participant explained: “she is not ill… she is different. We just need people to understand and accept her, not feel sorry for her.” (P4).

Parents also discussed inclusion in schools. They explained that placing a child in a regular classroom was not enough if appropriate understanding and support were not available. One mother explained: “Inclusion is not just about putting him in a regular classroom… what matters is having people there who understand how to deal with him.” (P7).

## 4. Discussion

The present study provides a qualitative account of how Saudi families experience FQOL while raising a child with autism. Across the five themes, FQOL emerged not as a fixed outcome or a collection of independent domains, but as an evolving process shaped by diagnostic experiences, the reorganization of caregiving and family roles, the responsiveness of formal services, the development of family knowledge and agency, and opportunities for participation within the wider social environment. This interpretation is consistent with FQOL frameworks that conceptualize family well-being as arising from interactions among family characteristics, supports, and environmental conditions [3,4]. It also aligns with recent quantitative evidence showing that FQOL among families of autistic children is associated with social support, parenting self-efficacy, family functioning, and contextual resources [8,16].

The contribution of the present findings lies in showing how these influences become interconnected in everyday family life. Rather than operating as isolated predictors, diagnosis, caregiving, services, adaptation, and community conditions appeared to influence one another over time. In this sense, the findings complement existing Saudi research that has largely examined child quality of life, caregiver quality of life, service satisfaction, or standardized FQOL domains separately [14,15,16].

### 4.1. Diagnosis as the Beginning of Family Reorientation

The findings suggest that diagnosis functioned as a process of family reorientation rather than as a discrete clinical event. Parents often recognized developmental differences before formal identification and described the period preceding diagnosis as one of uncertainty, repeated help-seeking, and difficulty in making decisions about intervention and education. This pattern is consistent with previous research showing that parents often identify developmental concerns before formal diagnosis and experience uncertainty, as well as emotional and informational needs, during the diagnostic period [23,24,25,26,27].

However, the present findings extend this literature by suggesting that the significance of diagnosis was not limited to resolving uncertainty or producing emotional distress. Diagnosis also prompted families to reconsider expectations, redefine caregiving roles, and move from questioning the diagnosis toward identifying practical ways to support their children. This progression is consistent with qualitative evidence describing parental adjustment, adaptation, advocacy, and reconstruction of family life [28,29].

From an FQOL perspective, early family adaptation may influence subsequent experiences with services, caregiving, and parental confidence. Longitudinal research has shown that FQOL can change following an autism diagnosis and that parenting self-efficacy and social support are positively associated with FQOL [8]. The present findings help explain how such relationships may develop, as increasing understanding of autism appeared to provide families with a clearer sense of direction and greater confidence in making decisions.

These findings are also relevant within the Saudi context. Previous Saudi research has shown that family support and the availability and quality of autism services influence child and family outcomes [14,19]. The present study adds an experiential perspective by showing that families may begin evaluating and navigating these supports from the earliest stages following diagnosis. Post-diagnostic services may therefore need to address not only intervention referral but also parents’ emotional adjustment, understanding of autism, and confidence in navigating available systems [30].

### 4.2. Caregiving and Family Reorganization

The second theme demonstrates that caregiving affected FQOL primarily through the reorganization of everyday family life. Rather than describing caregiving solely in terms of burden or parental distress, participants emphasized changes in routines, time use, employment, relationships, shared responsibilities, and future planning. This finding is consistent with qualitative and family-based research showing that raising an autistic child can reshape daily occupations and routines across the household [31,32,33]. More recent synthesis similarly indicates that families actively negotiate everyday routines and participation in response to the sensory, behavioral, and support needs of autistic children [34].

The present findings extend this literature by showing that such reorganization was not experienced solely as disruption. Families also described redistributing responsibilities, developing greater consistency between caregivers, and adjusting routines in ways that promoted stability. This distinction is important because an FQOL perspective draws attention to how families function collectively and adapt to changing demands. Reviews have documented effects of autism on parental and family functioning, including time pressures, financial demands, and reduced family well-being, while emphasising the importance of considering family-level alongside child outcomes [35].

This family-level interpretation is also supported by Saudi evidence. Alnahdi et al. [16] found differences across multiple FQOL dimensions, including family interaction, parenting, emotional well-being, and physical/material well-being. Saudi evidence has similarly shown lower quality of life across multiple domains among caregivers of children with ASD [36]. The present study adds depth to these quantitative domains by illustrating how they become intertwined in everyday decisions. Employment decisions were not made independently of caregiving, and shared responsibility formed part of how families maintained continuity and stability.

The findings regarding social and family support are also consistent with Saudi research demonstrating the protective role of social support in parental well-being. Khusaifan and El Keshky [12] found that perceived social support moderated and mediated associations between parental stress and life satisfaction among Saudi parents of autistic children. In the present study, however, support also operated at a practical family-system level, with responsibilities redistributed and caregiving strategies coordinated across family members. This suggests that interventions aimed at improving FQOL may benefit from considering the family unit rather than focusing exclusively on the psychological burden experienced by a single caregiver.

### 4.3. Responsive Services as a Determinant of Family Quality of Life

The third theme highlights the central role of service responsiveness in shaping family quality of life. Participants did not evaluate services simply according to whether they were available; rather, they judged their value according to quality, intensity, continuity, individualization, and observable developmental benefit. This distinction is particularly important in the Saudi context, where previous research has documented variation in service utilization according to geographic location, family income, parental education, and autism knowledge [11]. More recent Saudi evidence indicates that autism services have expanded over time, although parents continue to report variability in service quality and support [18,20].

The present findings add an important qualitative distinction to this literature. Families described service availability and service responsiveness as related but not interchangeable. A programme could be accessible yet still be experienced as inadequate when intervention intensity was insufficient, staff were poorly matched to the child’s needs, or services failed to adapt as the child developed. This is consistent with Almasoud and Alqahtani’s [19] qualitative study linking service quality to quality-of-life outcomes for autistic individuals and their families. The present study extends this finding by illustrating how families assessed responsiveness through changes in children’s everyday communication, behavior, independence, and participation.

A particularly noteworthy finding concerned discontinuity when children’s developmental needs changed. In some cases, progress reduced eligibility for specialized services without ensuring that mainstream educational environments were prepared to provide appropriate support. This suggests that developmental progress does not necessarily reduce the need for services but can alter the type of support required. Such experiences highlight the importance of continuity across service systems and flexible eligibility pathways rather than service models organized solely around static diagnostic categories [37].

The relationship between child progress and family well-being was also evident. Participants described meaningful developmental progress as reducing aspects of everyday caregiving demand and increasing parental confidence and hope. This is consistent with Alhuzimi’s [15] Saudi study identifying services, family support, child-related difficulties, and intervention experiences as factors associated with parents’ perceptions of autistic children’s quality of life. It also aligns with Almasoud and Alqahtani [19], who emphasized the relationship between service quality and quality of life. The present study adds a possible pathway through which this relationship operates: responsive services may promote developmental progress, which in turn can alter everyday family functioning and perceptions of FQOL.

Financial and geographic barriers further complicated this process. Participants described private expenditure as closely tied to attempts to secure services they considered effective, while families outside major urban centers faced additional barriers to specialized provision. These experiences are consistent with evidence of geographic and socioeconomic differences in autism service utilization in Saudi Arabia [11,38]. They reinforce the need to evaluate service systems not solely by the number of programs available, but by whether families can access sufficiently intensive, continuous, and developmentally appropriate support.

### 4.4. Knowledge, Agency, and Family Resilience

The fourth theme suggests that family adaptation was supported by the gradual development of knowledge, caregiving competence, confidence, and hope. Participants did not describe resilience as the absence of difficulty or distress. Rather, it appeared to develop through accumulated experience, greater understanding of autism, and increasing confidence in interpreting children’s behavior and making decisions about support.

This interpretation is consistent with contemporary research that conceptualizes resilience as a process involving adaptive resources rather than a fixed personal characteristic. Cardelle-Pérez et al. [9], for example, found meaningful relationships between resilience and FQOL among parents of autistic children while also highlighting the importance of adequate resources and support. Longitudinal evidence further indicates that parenting self-efficacy and social support are positively associated with FQOL among parents following an autism diagnosis [8]. Together, these findings support the interpretation that confidence and adaptive capacity can develop alongside family experience rather than simply reflecting pre-existing parental characteristics.

The present study adds a more experiential account of how this process may occur. Knowledge allowed some parents to reinterpret behaviors that had previously generated confusion or frustration, make more informed service decisions, and engage with professionals with greater confidence. In this sense, knowledge functioned not merely as information but as a source of parental agency. This interpretation also resonates with Saudi qualitative evidence identifying autism knowledge, parent training, and information as important needs among mothers of children with ASD [21].

Importantly, participants’ accounts also suggest that hope was grounded in observable experience. Developmental progress provided families with evidence that their efforts could produce meaningful change, thereby reinforcing confidence and future expectations. Rather than treating resilience and hope as individual psychological traits, the findings therefore suggest that they may be partly relational and experience-based, developing through interactions among family knowledge, service experiences, child progress, and available support.

This interpretation has implications for FQOL research. Quantitative studies can identify associations among resilience, self-efficacy, support, and family quality of life, but the present qualitative findings help illustrate how those associations may be produced in everyday life. Family resilience may therefore be strengthened not only through psychological coping interventions but also through accessible information, parent education, opportunities to develop caregiving competence, responsive professional partnerships, and services that allow families to observe meaningful progress.

### 4.5. Community Understanding, Participation, and Meaningful Inclusion

The final theme demonstrates that FQOL was influenced not only by family and service factors but also by the social environments in which families participated. Participants described misunderstanding, unsolicited parenting advice, and negative interpretations of autistic behavior as additional sources of emotional work. Conversely, greater understanding among relatives and community members made social participation easier and reduced the need for families to repeatedly explain or justify children’s behavior.

These experiences are consistent with Saudi evidence showing that stigma and discrimination can negatively affect parents’ experiences and quality of life [13,39]. Alshaigi et al. [13] found that a substantial proportion of parents of autistic children in Riyadh reported experiences of stigma, with mothers reporting particularly high levels of self- and enacted stigma. Recent research has also demonstrated that affiliate stigma is negatively associated with FQOL among parents of children with ASD and that parenting self-efficacy may help explain part of this relationship [40]. These findings support the view that community responses to autism can influence family well-being beyond the immediate characteristics of the child [41].

The present study extends this literature by highlighting participation as an important mechanism linking social understanding to FQOL. Families explained that the degree of understanding around them influenced whether they felt able to travel, attend family gatherings, visit relatives, or participate comfortably in community life. Thus, misunderstanding could extend beyond stigma itself to restrict ordinary family participation.

Participants also distinguished between sympathy and meaningful inclusion. This distinction was particularly evident in education, where physical placement in a mainstream classroom was not considered sufficient if teachers and school environments were unable to respond appropriately to the child’s needs [42]. This interpretation is consistent with broader FQOL perspectives in which family well-being is shaped by interactions with educational and community systems, rather than by household circumstances alone.

Informal support was similarly valued when it was responsive and informed. The presence of relatives or social networks was not automatically experienced as supportive; assistance became meaningful when others understood autism and adjusted their responses to the child and family. This complements Saudi evidence showing the protective role of perceived social support in parental well-being and suggests that the quality of social support may matter alongside its availability [12].

These findings position social inclusion as a family-level FQOL issue rather than solely an outcome for the autistic child. Efforts to improve community awareness may therefore need to move beyond providing general information about autism toward creating social, educational, and community environments in which autistic individuals and their families can participate without unnecessary explanation, judgment, or exclusion.

### 4.6. Practical Implications

The findings have several practical implications for autism services, education, and family support in Saudi Arabia. First, family support should begin at or shortly after diagnosis. Families may experience emotional stress as well as uncertainty about intervention, education, and available services. Support at this stage should therefore provide clear information, guidance in accessing services, and opportunities for families to build confidence in responding to their child’s needs. Second, autism services should be evaluated not only by their availability but also by how well they respond to children’s changing needs. Families valued high-quality intervention, sufficient intensity, continuity of support, good communication with professionals, and improvements in their child’s daily functioning. A family-centered approach should therefore consider both child outcomes and the well-being and functioning of the family. Third, better continuity is needed between specialized and mainstream services. Some families described difficulties when their children no longer qualified for specialized services but did not receive sufficient support in mainstream settings. This highlights the need for flexible transitions and better coordination between educational and therapeutic services. Finally, improving family quality of life requires attention beyond formal services. Educational inclusion should involve appropriate support, knowledgeable staff, and meaningful participation rather than placement alone. Community awareness should also focus not only on increasing knowledge of autism but on reducing stigma and creating environments in which autistic individuals and their families can participate comfortably and confidently.

### 4.7. Limitations and Future Research

Several limitations should be considered when interpreting the findings. First, the study included a relatively small number of participants, all of whom were recruited within the Saudi context. Although qualitative research seeks depth rather than statistical generalization, the experiences described may not represent all families of children with autism, particularly those living in different cultural or service contexts. Second, the findings reflect the perspectives of parents and one sibling caregiver. Including the perspectives of individuals with autism themselves and other healthcare professionals may provide a more comprehensive understanding of the factors shaping family quality of life. Third, longitudinal qualitative research would provide valuable insight into how adaptation and quality of life develop across different stages of the autism journey. Finally, conducting interviews through Zoom may have affected rapport and how openly participants shared their experiences. Future research should also examine how specific characteristics of intervention services—including accessibility—influence FQOL over time. Comparative studies across regions and countries may further clarify how cultural, educational, and healthcare systems contribute to differences in family experiences.

## 5. Conclusions

This study provides an in-depth understanding of how Saudi families experience and negotiate FQOL while raising an autistic child. The findings suggest that FQOL is not shaped by any single factor but emerges through ongoing interactions among family caregiving, access to responsive services, developing knowledge and adaptive capacity, and opportunities for meaningful social participation. Importantly, families’ accounts showed that challenges and adaptation could coexist: caregiving demands remained substantial, while knowledge, shared responsibility, effective support, and children’s developmental progress contributed to greater confidence and hope. The findings also highlight that the availability of services or opportunities for inclusion alone may be insufficient when they are not responsive to children’s changing needs and family circumstances. Supporting FQOL therefore requires a coordinated, family-centered approach that extends beyond the child to consider the well-being, resources, participation, and evolving needs of the family as a whole.

## Figures and Tables

**Table 1:** Participant characteristics.

Participant	Relation to Child	Child Age	Child Gender	Diagnosis Years
P1	Mother	7	Male	3
P2	Mother	8	Male	5
P3	Mother	6	Male	2.5
P4	Mother	12	Female	6
P5	Mother	8	Male (twins)	4
P6	Sister	24	Female	10
P7	Mother	10	Male	3
P8	Mother	6	Male	4
P9	Father	8	Male	4
P10	Father	7	Male	5
P11	Mother	6	Male	4
P12	Mother	10	Male	3

**Table 2:** Overview of Themes and Subthemes.

Theme	Subthemes
Theme 1. Diagnostic Experiences and Family Reorientation	Early Recognition of Developmental Differences Diagnostic Uncertainty and Delayed Identification Emotional Responses and Family Reorientation
Theme 2. Caregiving and Family Reorganization	Continuous Caregiving Responsibilities Family Roles and Shared Responsibility Balancing Caregiving with Personal and Family Life
Theme 3. Responsive Services and Family Quality of Life	Service Responsiveness and Perceived Effectiveness Changing Needs and Continuity of Support Barriers to Service Access and Financial Pressure Progress and Family Quality of Life
Theme 4. Family Adaptation Through Knowledge and Resilience	Acceptance as a Foundation for Adaptation Knowledge as a Source of Empowerment
Theme 5. Community Understanding and Social Inclusion	Public Understanding of Autism Community Participation and Support Meaningful Inclusion

## Data Availability

Access to the data is restricted due to privacy, confidentiality, and ethical considerations. The study involved qualitative interviews with parents and caregivers, and the interview transcripts contain sensitive and potentially identifiable information about participants and their families. Therefore, the full interview data cannot be made publicly available.

## Abbreviations

The following abbreviations are used in this manuscript:

ASD	Autism Spectrum Disorder
FQOL	Family quality of life

## References

[1] American Psychiatric Association (2022). Diagnostic and Statistical Manual of Mental Disorders.

[2] Hoffman L., Marquis J., Poston D., Summers J.A., Turnbull A. (2006). Assessing Family Outcomes: Psychometric Evaluation of the Beach Center Family Quality of Life Scale. J. Marriage Fam..

[3] Samuel P.S., Rillotta F., Brown I. (2011). Review: The development of family quality of life concepts and measures. J. Intellect. Disabil. Res..

[4] Zuna N.I., Turnbull A., Summers J.A. (2009). Family Quality of Life: Moving From Measurement to Application. J. Policy Pr. Intellect. Disabil..

[5] Losada-Puente L., Baña M., Asorey M.J.F. (2022). Family quality of life and autism spectrum disorder: Comparative diagnosis of needs and impact on family life. Res. Dev. Disabil..

[6] Schlebusch L., Dada S., Samuels A.E. (2017). Family Quality of Life of South African Families Raising Children with Autism Spectrum Disorder. J. Autism Dev. Disord..

[7] Wang R., Liu Q., Zhang W. (2022). Coping, social support, and family quality of life for caregivers of individuals with autism: Meta-analytic structural equation modeling. Pers. Individ. Differ..

[8] Dai Y., Chen M., Deng T., Huang B., Ji Y., Feng Y., Liu S., Zhang L. (2023). The importance of parenting self-efficacy and social support for family quality of life in children newly diagnosed with autism spectrum disorder: A one-year follow-up study. Autism Res..

[9] Cardelle-Pérez F., Banacloig-Delgado C., García-García L. (2023). Resilience and family quality of life in parents of children with autism spectrum disorders. Clin. Child Psychol. Psychiatry.

[10] Lei X., Kantor J. (2020). Social support and family quality of life in Chinese families of children with autism spectrum disorder: The mediating role of family cohesion and adaptability. Int. J. Dev. Disabil..

[11] Alnemary F.M., Aldhalaan H.M., Simon-Cereijido G., Alnemary F.M. (2016). Services for children with autism in the Kingdom of Saudi Arabia. Autism.

[12] Khusaifan S.J., Keshky M.E.S.E. (2021). Social Support as a Protective Factor for the Well-Being of Parents of Children with Autism in Saudi Arabia. J. Pediatr. Nurs..

[13] Alshaigi K., Albraheem R., Alsaleem K., Zakaria M., Jobeir A., Aldhalaan H. (2020). Stigmatization among parents of autism spectrum disorder children in Riyadh, Saudi Arabia. Int. J. Pediatr. Adolesc. Med..

[14] Salami S., Alhalal E. (2024). Gender differences in predictors of quality of life for parents of children with Autism Spectrum disorder in Saudi Arabia. J. Pediatr. Nurs..

[15] Alhuzimi T.E. (2024). Saudi parents’ perspectives of the factors influencing the quality of life of their children with autism spectrum disorder. Res. Dev. Disabil..

[16] Alnahdi G.H., Alwadei A., Schwab S. (2024). Family quality of life of caregivers of individuals with autism, with other disabilities, and without disabilities: The case of Saudi Arabia. Int. J. Dev. Disabil..

[17] Alodaili M.S.M., Cornejo L.T.O., Saleh K.A., Alrashedi H., Alrubaiee G.G., Almoliky M.A., Alqalah T.A.H., Alkubati S.A. (2025). Quality of life of caregivers of children with autism spectrum disorder in Saudi Arabia. J. Pediatr. Nurs..

[18] Almasoud H., Ain G. (2023). Parental perspectives on autism services in Saudi Arabia: Decade comparison (2011–2021). Res. Dev. Disabil..

[19] Almasoud H., Alqahtani S. (2023). Potential impact of autism services on the quality of life of individuals with autism spectrum disorder and their families. Res. Dev. Disabil..

[20] Saleh S.R., Alaoufi H.M., Eltantawy M.M. (2024). Parental satisfaction with the quality of services provided to persons with autism spectrum disorder and their families in Saudi Arabia. Front. Psychiatry.

[21] Hemdi A., Daley D. (2017). The needs of mothers of children with autism spectrum disorder (ASD) in the Kingdom of Saudi Arabia (KSA): A qualitative study. Int. J. Acad. Sci. Res..

[22] Braun V., Clarke V. (2006). Using thematic analysis in psychology. Qual. Res. Psychol..

[23] Crane L., Chester J.W., Goddard L., A Henry L., Hill E. (2015). Experiences of autism diagnosis: A survey of over 1000 parents in the United Kingdom. Autism.

[24] Boshoff K., Gibbs D., Phillips R.L., Wiles L., Porter L. (2018). A meta-synthesis of how parents of children with autism describe their experience of advocating for their children during the process of diagnosis. Heal. Soc. Care Community.

[25] Makino A., Hartman L., King G., Wong P.Y., Penner M. (2021). Parent Experiences of Autism Spectrum Disorder Diagnosis: A Scoping Review. Rev. J. Autism Dev. Disord..

[26] Smith-Young J., Chafe R., Audas R. (2020). “Managing the Wait”: Parents’ Experiences in Accessing Diagnostic and Treatment Services for Children and Adolescents Diagnosed With Autism Spectrum Disorder. Heal. Serv. Insights.

[27] Goh J.X., Aishworiya R., Ho R.C.M., Wang W., He H. (2021). A qualitative study exploring experiences and support needs of parents of children with autism spectrum disorder in Singapore. J. Clin. Nurs..

[28] Ooi K.L., Ong Y.S., Jacob S.A. (2016). A meta-synthesis on parenting a child with autism. Neuropsychiatr. Dis. Treat..

[29] Corcoran J., Berry A., Hill S. (2015). The lived experience of US parents of children with autism spectrum disorders. J. Intellect. Disabil..

[30] Myers L., Karp S.M., Dietrich M.S., Looman W.S., Lutenbacher M. (2020). Family-Centered Care: How Close Do We Get When Talking to Parents of Children Undergoing Diagnosis for Autism Spectrum Disorders?. J. Autism Dev. Disord..

[31] DeGrace B.W. (2004). The Everyday Occupation of Families With Children With Autism. Am. J. Occup. Ther..

[32] DePape A.-M., Lindsay S. (2014). Parents’ Experiences of Caring for a Child With Autism Spectrum Disorder. Qual. Health Res..

[33] Schaaf R.C., Toth-Cohen S., Johnson S.L., Outten G., Benevides T.W. (2011). The everyday routines of families of children with autism: Examining the impact of sensory processing difficulties on the family. Autism.

[34] Daly G., Jackson J., Lynch H. (2022). Family life and autistic children with sensory processing differences: A qualitative evidence synthesis of occupational participation. Front. Psychol..

[35] Karst J.S., Van Hecke A.V. (2012). Parent and Family Impact of Autism Spectrum Disorders: A Review and Proposed Model for Intervention Evaluation. Clin. Child Fam. Psychol. Rev..

[36] Al-Jabri B.A., Abualhamael R.M., Al Hazza M.T., Bahabri S.A., Alamri Y.M., Alghamdi B.M. (2022). Quality of life of caregivers of autistic children in Saudi Arabia: Cross-sectional study. Neurosciences.

[37] Mello C., Rivard M., Patel S., Morin D., Morin M. (2022). Assessing the quality of care and service trajectories in autism from families’ perspective: Early intervention and interim services. Res. Dev. Disabil..

[38] Wallace-Watkin C., Sigafoos J., Waddington H. (2022). Barriers and facilitators for obtaining support services among underserved families with an autistic child: A systematic qualitative review. Autism.

[39] A AlHefdhi H., AL Zomia A.S., Alshehri N.M., A Alaskari A., A Hussain A., A Lahiq L., A Asiri M., Al Asiri W., Alahmari A.M., Asiri H.M. (2024). Examining the Quality of Life and Discrimination Impact on Parents of Children With Autism Spectrum Disorder in Aseer Region, Saudi Arabia: A WHO-QOL Survey. Cureus.

[40] Ye Y., Deng T., Chen M., Huang B., Ji Y., Feng Y., Liu S., Zhang T., Zhang L. (2024). Relationship between affiliate stigma and family quality of life among parents of children with autism spectrum disorders: The mediating role of parenting self-efficacy. Arch. Psychiatr. Nurs..

[41] Kinnear S.H., Link B.G., Ballan M.S., Fischbach R.L. (2015). Understanding the Experience of Stigma for Parents of Children with Autism Spectrum Disorder and the Role Stigma Plays in Families’ Lives. J. Autism Dev. Disord..

[42] McKinlay J., Wilson C., Hendry G., Ballantyne C. (2022). “It feels like sending your children into the lions’ den”—A qualitative investigation into parental attitudes towards ASD inclusion, and the impact of mainstream education on their child. Res. Dev. Disabil..

